# Immersive robotic colorectal training in the United Kingdom is safe and efficient

**DOI:** 10.1308/rcsann.2024.0105

**Published:** 2025-07-24

**Authors:** J Franklyn, P Vaughn Shaw, M Abdalkoddus, R Coates, S Holtham, G Farook

**Affiliations:** ^1^ West Hertfordshire Teaching Hospitals NHS Trust, UK; ^2^ NHS Lothian, UK; ^3^ University Hospitals Plymouth NHS Trust, UK; ^4^ South Tyneside and Sunderland NHS Foundation Trust, UK

**Keywords:** Robotic, Colorectal

## Abstract

**Introduction:**

With increased utilisation of robotics in surgery, the demand for structured training is increasing. This paper presents the safety and efficacy profile of a robotic colorectal fellowship in the United Kingdom.

**Methods:**

This is a retrospective study conducted in a district general hospital between 2019 and 2023. Procedures performed using the Da Vinci robot were divided into two cohorts, those performed by robotic fellows and those performed by consultant surgeons. Each fellowship lasted on average six months and at the end of the fellowship the trainee had completed the mandatory requirements to be certified as a robotic surgeon. The objective of this study is to compare the outcomes of procedures performed by the fellows with those performed by consultants.

**Results:**

Of the 224 robotic procedures recorded, 117 were performed by consultants and 107 by fellows. The median time to complete the procedure was 315min vs 257min for consultants and fellows, respectively. The average postoperative length of stay was 6 and 7 days, the anastomotic leak rate was 4.3% and 5.6% and reoperation rate was 11% and 9% for procedures performed by consultants and fellows, respectively. The median lymph nodal yield was 17 in both groups and the margin positivity rate (R1) resection rate was 7% and 4% (*p* = 0.4) for procedures performed by consultants and fellows.

**Conclusion:**

It is possible to safely train in robotic colorectal surgery without compromising patient safety, oncological outcomes or adversely affecting theatre efficiency.

## Introduction

Robotic surgery is poised to surpass laparoscopy as the preferred minimally invasive modality by which to perform proctectomies and colectomies by 2025, signalling a transformational shift towards robotics.^1^ Given the potential advantages of robotic-assisted surgery over conventional laparoscopy, the appetite to expand the robotic colorectal service is significant; however, a training bottleneck has emerged.^2–4^ At present, there are predominantly two pathways to achieve robotic competence in the United Kingdom (UK): first, as a consultant via the proctorship programme; and second, as a trainee, either during specialty training or after completion of training during a fellowship. Irrespective of the pathway used, training in robotic colorectal surgery is not entirely streamlined, resulting in widespread frustration among surgical trainees.^5–8^ To match the demand for robotic-assisted surgery, a streamlined conveyor belt of trainees who are competent in robotic surgery at the end of their training/fellowship is crucial. This paper presents evidence for and the safety profile of an immersive robotic colorectal fellowship programme in the north-eastern region of the UK and its implications for wider robotic colorectal training.

## Methods

This is a retrospective study of a prospectively maintained database in a district general hospital in the north-eastern part of the UK. The hospital performs 150 colorectal resection per year including approximately 40 rectal resections per year. Robotic colorectal surgery was introduced in early 2015 in the trust.

### Inclusion criteria

All procedures performed on a dual console Da Vinci Xi^®^ robot between 2019 and 2023 were recorded and divided into two cohorts. The first cohort involved procedures performed by consultant colorectal surgeons. All procedures performed by consultants between 2019 and 2023 were included. The second cohort involved procedures performed predominantly by robotic fellows under the supervision of robotic trainers between 2021 and 2023. During this period there were four trainees; each fellowship lasted on average 6 months (range 5–7 months). At the end of the fellowship, trainees had completed the mandatory requirements to be certified as a competent, independent console surgeon and were all appointed as robotics-trained colorectal consultants.

### The trainers and robotic fellows

A robotic colorectal service in the hospital was initiated in 2015 and consultant surgeons performed more than 100 procedures prior to 2019. This period was excluded from analysis because the consultants were deemed to be in their learning curve. Between 2019 and 2021, the procedures were predominantly performed by consultant surgeons with assistance from registrars. Over a two-year period between 2021 and 2023, four post-Certification of Completion of Training (CCT) fellows (trainees who have completed their training or CCT) were appointed as robotic fellows. All four fellows had limited exposure to structured robotic training before starting the fellowship. Two of the four robotic fellows did not have any formal training in robotics during their formative speciality training; the other two had experience in bedside assisting of robotic cases and had completed their simulation requirements before starting their fellowship.

### Fellowship programme

At any point, only one fellow was employed by the hospital to concentrate training resources and opportunities. The robotic fellow had access to two colorectal robotics lists weekly and at least one urology list a week to provide additional exposure to robotic pelvic surgeries such as prostatectomy and urinary bladder surgery. All procedures performed by the fellow were overseen by the robotics proctor. A bespoke training pathway was followed based on the fellow’s previous robotics experience. A one-size-fits-all approach was not followed. It was generally agreed that trainees start by being proficient in bedside assistance. As per the European Academy of Robotic Colorectal Surgeons (EARCS) proficiency guidelines, the surgical procedure was broken down into small self-contained units or modules. For example, the modules for anterior resection included positioning, port placement, set-up and independently docking the robot. These were followed by inferior mesenteric artery and vein ligation, total mesorectal excision and finally splenic flexure mobilisation where appropriate. By the end of the fellowship, the fellow was able to piece together these modules and complete the entire resection independently. The objective was to ensure that all fellows were safe and competent independent robotic surgeons. Assessment of competence was both qualitative and quantitative as per the EARCS and intuitive criteria.^9,10^

### Competence assessment and requirements

To be deemed competent, a trainee was expected to have completed simulation-based training on the Da Vinci Xi^®^, online competency assessments, dry lab training, have assisted in at least 10 bedside procedures and have performed at least 20 procedures as the primary surgeon. All aspects of surgical robotic training from theatre set-up, port placement, docking the robot and completion of the entire surgical procedure were logged and critically appraised.^10^

The primary objective of this study was to compare the safety and efficacy of procedures performed by the fellows against those performed by consultants. This involved computing the average blood loss, operating time, major perioperative complications and conversion to open surgery. Anastomotic leaks were defined as any radiological or clinical leak, irrespective of whether the leak warranted intervention.

Secondary outcomes involved assessing the short-term oncological outcomes between the two groups. This involved analysis of the total lymph node yield, circumferential resection margin and distal margin involvement (R1).

We used the American Joint Committee on Cancer (AJCC) 7th edition TNM classification to stage cancers. The study was reported in accordance with STROBE guidelines, and anonymised retrospective data were utilised.

### Ethics approval

As a retrospective observational study approved by the clinical governance department as an ongoing service evaluation project this study was exempt from ethics approval and informed consent was not considered necessary.

### Statistical analysis

Categorical variables are presented as frequencies and percentages and were compared using the chi-squared test or Fisher’s exact test (with Bonferroni adjustment for multiple testing and post-hoc analysis when needed) as appropriate. Continuous variables are presented as medians and interquartile range (IQR) and were compared Wilcoxon rank-sum test or Kruskal–Wallis test as appropriate. A *p* value of ≤0.05 was considered statistically significant. Statistical analysis was performed using R software (version 4.0.4, R Foundation for Statistical Computing, Vienna, Austria).

## Results

Between 2019 and 2023 a total of 224 robotic procedures were performed. The majority of operations performed during the study period were either an anterior resection or abdominoperineal excision. The median age of patients was 66.5 years (IQR 58–74) with a male to female ratio of 2:1. The median body mass index of the patient cohort was 27kg/m^2^ (IQR 24–31). Eleven patients (5%) suffered anastomotic leak, 21 (9%) needed reoperation, and 27 (12%) were readmitted within 30 days. The median hospital stay for the cohort was 6 days (IQR 4–11). Margin positivity (R1 resection) was present in 13 patients (6%) with a median lymph node yield of 17 (IQR 13–23). Table 1 summarises the baseline data of all procedures performed.

**Table 1:** Overall patient demographics and clinical outcomes between 2019 and 2023

Variable	Outcome (*N* = 224)
Age, years (IQR)	66.5 (58–74)
Sex (M:F)	148:76 (2:1)
ASA grade (median)	2
Body mass index	27 (24–31)
Length of stay (days)	6 (4–11)
Blood loss >100ml	26 (13)
Conversion to open	2 (0.89)
Margin positivity	13 positive (6)
Lymph nodal yield	17 (13–23)
Preoperative radiotherapy	32 (14)
Anastomotic leak	11 (5)
Reoperation	21 (9)
Readmission within 30 days	27 (12)

Values are given as median (IQR) or *n* (%).

ASA = American Society of Anesthesiologists; F = female; IQR = interquartile range; M = male

Consultants performed 117 of these procedures and 107 procedures were performed by robotic colorectal fellows under the direct supervision of consultant surgeons. Tables 2 and 3 compare case demographics and outcomes when the procedures were performed by consultants and fellows. Both groups had comparable baseline demographics. Procedures performed by fellows had significantly shorter operating times. There was significantly increased blood loss in cases performed by fellows but all other postoperative and oncological outcomes were similar in the two groups. Subgroup analyses of individual fellows’ case load and clinical outcomes revealed no difference in either case selection or clinical outcomes between individual fellows (Table 4).

**Table 2:** Break-up of standard procedures performed by the consultants and the fellows during the study period with a comparison of operation durations

Operation	Consultant	Fellow	*p* value
Anterior resection (*n*) Time, minutes	75 315 (250–389)	69 257 (215–321)	**0.003**
APR (*n*) Time, minutes	28 301 (283–363)	27 328 (289–383)	0.3

Values are given as *n* or median (IQR)

APR = abdominoperineal resection

**Table 3:** Comparison of procedures’ outcomes between the consultant surgeons and the robotic fellows

	Consultant (*N* = 117)	Fellow (*N* = 107)	*p* value
Age, years	67 (60–74)	64 (57–73)	0.3
Sex (M:F)	1.9:1	2.1:1	0.5
ASA grade			0.1
1	7 (6)	7 (7)	
2	84 (72)	63 (59)	
3	26 (22)	37 (35)	
BMI, kg/m^2^	27 (24–31)	27 (25–31)	0.65
Length of stay, days	7 (5–11)	6 (4–11)	0.2
Blood loss >100ml	7 (7)	19 (19)	**0.02**
Conversion to open	2 (1.7)	0	
Margin positivity	9 (8)	4 (4)	0.4
AJCC stage			0.2
1	20 (21)	27 (34)	
2	37 (39)	27 (34)	
3	34 (36)	25 (32)	
4	2 (2)	0 (0)	
Lymph nodal yield	17 (13–24)	17 (13–22)	0.4
Preoperative radiotherapy	23 (23)	9 (11)	0.07
Anastomotic leak	5 (4.3)	6 (5.6)	0.9
Reoperations	13 (11)	8 (9)	0.7
Clavien–Dindo score			0.6
Readmissions	17 (15.6)	10 (11.6)	0.5

Values are given as median (IQR) or *n* (%).

AJCC = American Joint Committee on Cancer; ASA = American Society of Anesthesiologists; BMI = body mass index; F = female; IQR = interquartile range; M = male

**Table 4:** Comparison of individual outcomes among the four robotic fellows (number of procedures per fellow in brackets)

Variable	Fellow 1 (25)	Fellow 2 (35)	Fellow 3 (25)	Fellow 4 (22)	*p* value
Age, years	65 (57–74)	62 (56–71)	66 (56–72)	66 (61–74)	0.7
Sex (M:F)	1.8:1	2.2:1	3.8:1	1.4:1	0.5
BMI, kg/m^2^	27 (25–29)	27 (25–32)	28 (25–31)	29 (22–33)	1
Length of stay, days	6 (4–11)	7 (4–12)	7 (4–11)	6 (4–9)	0.8
Margin positive	0 (0)	3 (9)	1 (3)	0 (0)	0.3
AJCC stage					
3	7 (37)	8 (32)	8 (40)	2 (13)	**0.03**
2	5 (26)	13 (52)	2 (10)	7 (47)	
1	7 (37)	4 (16)	10 (50)	6 (40)	
LN number	17 (12–19)	17 (13–25)	16 (12–19)	17 (14–24)	0.4
Leak	2 (8)	2 (6)	0 (0)	2 (9)	0.5
Reoperation	1 (4)	4 (11)	2 (10)	1 (8)	0.8
Preoperative radiotherapy	5 (24)	2 (8)	1 (5)	1 (7)	0.3
Readmission	3 (12)	4 (13)	3 (14)	0 (0)	0.8

Values are given as median (IQR) or *n* (%).

AJCC = American Joint Committee on Cancer; BMI = body mass index; F = female; IQR = interquartile range; LN = lymph node; M = male

## Discussion

In a structured, ring-fenced training environment, it is possible to train senior fellows safely and efficiently in robotic colorectal surgery. Given the advantages of robotic surgery in shortening the postoperative recovery period and its advantages in pelvic surgery, there is significant interest in robotics within the surgical community.^2,4^ This has resulted in an increased desire among trainees to train in robotic colorectal surgery; unfortunately, the lack of structured training programmes in all regions of the country has led to increased frustration among the trainees.^7,8,11,12^ To meet this demand for robotics training, it is important to have a well-coordinated and efficient training programme. The fundamental advantage of a high-intensity training programme is the rapid upskilling of trainees irrespective of previous robotic experience, thereby equipping them to step into independent robotic practice with confidence. Recent evidence in robotic hernia repairs and robotic prostatectomies suggests that frequent practice is as important as case volume. Fast initial case acquisition is directly related to better training outcomes as opposed to doing a similar number of cases over a protracted period.^13^ Similar strategies have been employed in endoscopy training with the success of immersive colonoscopy training.^14,15^

Although we agree that case selection is important for trainees, the strength of an immersive training pathway is that senior fellows (who are likely to take on a consultant role imminently) are trained in a real-world setting under supervision, rather than cherry-picking cases over a year or two.^16^ The break-down of cases performed by consultant surgeons and fellows was similar, as depicted in Table 2. A reasonable number of complex rectal resections, such as patients with previous chemoradiotherapy, were also performed by fellows. The median body mass index of patients having colorectal robotic surgery in both study groups was similar at 27kg/m^2^.

The median time to complete the robotic procedure was significantly lower during the fellowship period (Table 3). Having a dedicated fellow in the theatre who is at a post-CCT level is akin to having dual-consultant operating lists, and all aspects of the surgical procedure from stoma formation, port insertion and other non-robotic steps are likely to be more efficient. However, it is also possible that the consultants would be more involved in challenging cases, thereby skewing the data. Irrespective of the reason, the fear that robotic surgical training will slow a procedure is unjustified because the combined expertise of having a consultant and a regular fellow probably improves efficiency and theatre utilisation.

The safety of a surgical procedure is as important as the efficiency. Despite having statistically significantly more blood loss, there were no incidents of major primary haemorrhage or reactionary haemorrhage when the procedure performed by a robotic fellow warranted conversion to open surgery. The length of postoperative stay was marginally better for procedures performed by fellows, although this was not statistically significant. Procedures performed by the fellows did not result in higher leak rates or have more incidents of reoperation for complications. The short-term oncological outcomes between the two groups were similar, and the positive margin resection rate (distal and circumferential) and the lymph nodal yield were similar between the two groups.

Ring-fenced robotic training, although useful for the training of post-CCT fellows, can be detrimental to doctors in training in their formative years. This can result in frustration and resentment among them. Recent reports have suggested that almost two-thirds of trainees felt that the introduction of robotics has had a negative effect on their training.^17^ The ability to balance the robotic training needs of registrars, fellows and consultants is a real-world challenge. The need to embed systematic robotic training into the surgical curriculum is an ongoing discussion within the Royal College of Surgeons of England. Ultimately with a wider availability of surgical robots, a revamped general surgical curriculum similar to other healthcare systems such as in the USA may help address the issue in the long run.^6,18–20^

Nevertheless, in the current era with our existing training pathways and difficulties in obtaining regular robotic access to train, the implication for the immersive colorectal robotic training model is significant. Identifying regional centres of expertise in robotic surgery and having senior trainees or fellows performing immersive training is a foolproof way of training competent robotic surgeons. At a national level, this method of training can help reduce the number of consultants taking on proctorship-based training as this could significantly impact the patient waiting lists and adversely affect surgical training of other trainees.^12,21,22^

### Study limitations

This is a single-centre, retrospective study and there are biases associated with such an analysis. Because the purpose of this paper was to analyse the safety and efficiency of an immersive robotic training programme, we have not reported the global assessment scores of the trainees to chart their competence progress. The intuitive Da Vinci Xi^®^ guidelines was used to define competence.^6^

## Conclusion

Immersive robotic colorectal training is safe and feasible in the UK. It is possible to safely train in robotic colorectal surgery irrespective of prior experience in a short time frame without compromising patient safety, oncological outcomes or adversely affecting theatre efficiency.
